# Protein Quality Control in the Endoplasmic Reticulum and Cancer

**DOI:** 10.3390/ijms19103020

**Published:** 2018-10-03

**Authors:** Hye Won Moon, Hye Gyeong Han, Young Joo Jeon

**Affiliations:** 1 Department of Biochemistry, Chungnam National University College of Medicine, Daejeon 35015, Korea; ansdj831@naver.com (H.W.M.); hanhk0123@naver.com (H.G.H.); 2 Department of Medical Science, Chungnam National University College of Medicine, Daejeon 35015, Korea

**Keywords:** endoplasmic reticulum (ER) stress, unfolded protein response (UPR) of the ER, ER-associated protein degradation (ERAD), autophagy, protein quality control, proteostasis, cancer

## Abstract

The endoplasmic reticulum (ER) is an essential compartment of the biosynthesis, folding, assembly, and trafficking of secretory and transmembrane proteins, and consequently, eukaryotic cells possess specialized machineries to ensure that the ER enables the proteins to acquire adequate folding and maturation for maintaining protein homeostasis, a process which is termed proteostasis. However, a large variety of physiological and pathological perturbations lead to the accumulation of misfolded proteins in the ER, which is referred to as ER stress. To resolve ER stress and restore proteostasis, cells have evolutionary conserved protein quality-control machineries of the ER, consisting of the unfolded protein response (UPR) of the ER, ER-associated degradation (ERAD), and autophagy. Furthermore, protein quality-control machineries of the ER play pivotal roles in the control of differentiation, progression of cell cycle, inflammation, immunity, and aging. Therefore, severe and non-resolvable ER stress is closely associated with tumor development, aggressiveness, and response to therapies for cancer. In this review, we highlight current knowledge in the molecular understanding and physiological relevance of protein quality control of the ER and discuss new insights into how protein quality control of the ER is implicated in the pathogenesis of cancer, which could contribute to therapeutic intervention in cancer.

## 1. Introduction

The endoplasmic reticulum (ER) is a dynamic and specialized membranous network of elongated tubules and flattened discs, and it spans a great area of the cytoplasm in the form of connected sacs and branching tubules [1]. The ER is involved in various cellular processes, including the biosynthesis of lipid species such as cholesterol, triacylglycerol, and phospholipids, the degradation of glycogen, detoxification, and the maintenance of Ca^2+^ homeostasis [2,3,4]. Intriguingly, the ER is in contact with every other cellular organelle and, in coordination, with these organelles exerts its multifaceted roles to sense intrinsic and extrinsic perturbations, combines stress signals, and manages cellular processes, indicating its role as a central coordinator for the maintenance of cellular homeostasis [5]. For example, the ER is physically interconnected with mitochondria and plays a pivotal role in the control of Ca^2+^ homeostasis. Additionally, the ER is in contact with plasma membrane [6]. The ER also associates with endosomes and contributes to cholesterol maintenance in endosomes [7,8]. Interestingly, the ER is in contact with the phagophore and eventually forms a mature autophagosome, suggesting its involvement in autophagy [9]. Most importantly, the ER on its own or in concert with other cellular organelles is involved in the biosynthesis, folding, assembly, and trafficking of secretory and transmembrane proteins, which constitute about one-third of all the proteins that are synthesized in the cell, indicating that the ER participates in important cellular and organismal processes involving protein degradation, signal transduction, lipid metabolism, and cell–cell communications [3,4]. 

**Figure 1:**
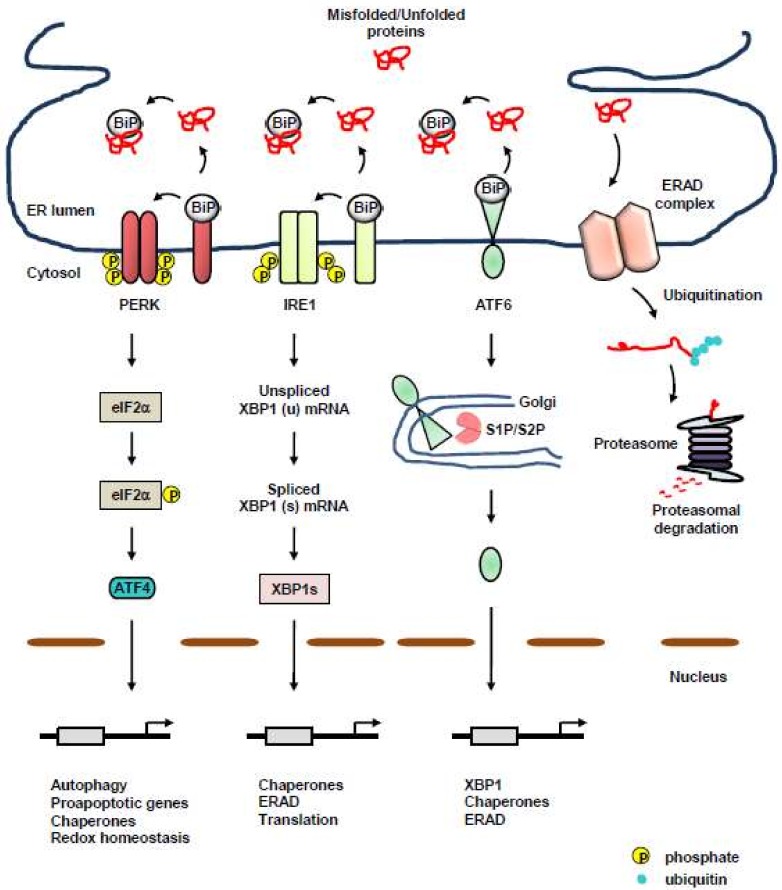
Protein quality-control machineries of the endoplasmic reticulum (ER). Protein quality-control machineries of the ER consist of three axes: acceleration of adequate protein folding, activation of the unfolded protein response (UPR), and protein clearance via ER-associated degradation (ERAD), or autophagy. UPR is composed of three transmembrane ER-resident stress sensors, inositol-requiring protein 1 (IRE1), activating transcription factor 6 (ATF6), and protein kinase RNA (PKR)-like ER kinase (PERK). Under unstressed conditions, the luminal domains of these UPR sensors are kept inactive via binding to a chaperone, binding immunoglobulin protein (BiP). Upon ER stress, BiP dissociates from the ER sensors, leading to the activation of UPR. The PERK/ATF4 axis induces the expression of chaperones and genes involved in autophagy, apoptosis, and redox homeostasis. The IRE1/X-box binding protein 1 (XBP1) axis facilitates the transcription of a subset of UPR genes linked to adequate folding and secretion of proteins, as well as ERAD [23,24,25,26]. Activated ATF6 induces the expression of chaperones, XBP1, and genes involved in ERAD. ERAD is also the protein quality-control machinery of the ER for removing terminally misfolded, unassembled, or tightly regulated proteins via the cytosolic ubiquitin–proteasome system (UPS). Following retrotranslocation across the ER membrane, ERAD substrates are ubiquitinated and degraded by the proteasome in the cytoplasm. Black arrow, facilitation; red line, misfolded/unfolded protein.

Protein quality-control machineries of the ER are composed of three axes: acceleration of adequate protein folding, activation of the unfolded protein response (UPR), and protein clearance via ER-associated degradation (ERAD), or autophagy [10,11] (Figure 1). Accumulation of misfolded or unassembled proteins, termed as ER stress, results in the activation of UPR to determine cell fate and function, subsequently restoring protein homeostasis, which is referred to as proteostasis. Even with the assistance of dedicated protein-folding machinery in the ER, a large portion of proteins entering the ER fail to obtain proper conformation due to physiological and pathological perturbations, and eventually, must be cleared [12,13]. Eukaryotic cells evolved ERAD for clearance of misfolded, unassembled, or tightly regulated proteins [14,15,16,17]. Furthermore, autophagy began to emerge as an another protein clearance mechanism of the ER to eliminate misfolded proteins for maintaining proteostasis [14,18]. Most importantly, a failure in the maintenance of proteostasis is closely associated with various protein misfolding diseases, such as cancer [19,20,21,22], implicating the importance of stringent protein quality-control machineries in the ER.

In this review, we not only highlight current knowledge in the molecular understanding and physiological relevance of ER stress and protein quality-control machineries of the ER, but also discuss new insights into how the adaptive protein quality-control capacity of the ER to pathophysiological perturbations is implicated in the modulation of malignancy, the regulation of cancer immunity, and the efficacy of therapies for cancer.

## 2. Protein Quality-Control Machineries of the ER: Three Transmembrane ER-Resident Stress Sensors of the UPR

Numerous extrinsic perturbations, such as accumulation of acidic waste, hypoxia and nutrient deprivation, and intrinsic perturbations (e.g., activation of oncogenes, inactivation of tumor-suppressive genes, accelerated secretion, and alteration in chromosome number), can disrupt the ER protein folding environment, resulting in the accumulation of misfolded proteins in the ER, which activates UPR. UPR of the ER is an interconnected signaling network that transduces the protein-folding status from the ER to the cytosol and nucleus, thereby reducing the load of secretory proteins and facilitating the folding of proteins. In multicellular eukaryotes, UPR consists of three transmembrane ER-resident UPR sensors, activating transcription factor 6 (ATF6) α and β, inositol-requiring protein 1 (IRE1) α and β, and protein kinase RNA (PKR)-like ER kinase (PERK) [27]. Under normal conditions, the luminal domains of these transmembrane ER-resident stress sensors are maintained inactive through the association with a chaperone, binding immunoglobulin protein (BiP; also known as GRP78), which belongs to the heat shock protein 70 family [28]. Above a critical threshold of misfolded and unfolded protein accumulation, BiP dissociates from the ER sensors and is recruited to misfolded proteins, resulting in the priming of all sensors for activation [29,30,31]. Protein disulfide isomerases (PDIs) are also suggested to modulate the ER sensors [32,33,34], raising the possibility of a combinational control for the activation of UPR. Interestingly, unfolded and/or misfolded proteins themselves can directly associate with IRE1 or PERK, thereby leading to dimerization, oligomerization, and subsequent activation of UPR [27,35,36,37]. However, if this adaptive UPR is overwhelmed by sustained ER stress, the cellular response switches from pro-survival to pro-death via the production of reactive oxygen species (ROS), the upregulation of pro-apoptotic B-cell lymphoma 2 (Bcl-2) family members, the regulation of microRNAs, and the release of Ca^2+^ [38].

### 2.1. PERK

PERK is an ER-resident transmembrane protein possessing a luminal stress-sensing domain and a cytosolic kinase domain [39]. Upon ER stress, PERK homodimerizes and trans-autophosphorylates to activate its cytoplasmic kinase domain [40]. The activated PERK in turn phosphorylates the α subunit of eukaryotic translation initiation factor 2 (eIF2α) at serine 51, which inhibits guanine nucleotide exchange factor (eIF2B) and lowers global messenger RNA (mRNA) translation, thereby reducing the load of newly synthesized proteins on the folding machinery of the ER and facilitating the cell to resolve the ER stress [39]. While the downstream response of UPR activation is a transient attenuation of global protein synthesis, certain species of mRNA are favorably translated, involving activation transcription factor 4 (ATF4; also known as CREB2), which transactivates various genes, including CCAAT/enhancer-binding protein (C/EBP) homologous protein (*Chop*), ER oxidoreductin 1 (*Ero1*), and growth arrest and DNA damage-inducible protein (*Gadd34*), all of which are involved in protein folding, autophagy, redox homeostasis, amino-acid metabolism, and apoptosis [41,42,43]. Interestingly, upregulated GADD34 plays a role as a cofactor of type 1 protein serine/threonine phosphatase (PP1) that dephosphorylates eIF2α, which creates a negative feedback loop for PERK signaling and restores protein synthesis [43,44,45]. CHOP plays a pivotal role in ER stress-induced apoptosis under excessive and chronic activation of PERK [43,46]. At initial time points after ER stress, transcription of *Chop* is suppressed several ways, including histone methylation and Toll-like receptor (TLR) signaling [47,48]. However, if ER stress is prolonged, ATF4 and CHOP function together as a heterodimer, which increases protein synthesis, protein misfolding, and oxidative stress, thereby leading to apoptosis [22,49].

### 2.2. IRE1

IRE1 is the most conserved ER stress sensor [50]. IRE1 possesses serine/threonine kinase activity and endoribonuclease activity within the cytoplasmic domain [50]. Upon sensing ER stress, released IRE1 from BiP dimerizes, oligomerizes, and trans-autophosphorylates, resulting in a conformational change that activates its RNase domain. Interestingly, an ER chaperone, heat shock protein 47 (Hsp47) is known to facilitate IRE1 signaling by binding to the luminal domain of IRE1, thereby blocking the association with BiP and subsequently promoting the oligomerization of IRE1 [51,52]. Additionally, nonmuscle myosin IIB (NMIIB) is shown to be required for oligomerization and activation of IRE1α [53]. Protein tyrosine phosphatase 1 B (PTP1B) also has an essential role in potentiating IRE1-mediated ER stress signaling [54]. Furthermore, ER-resident protein disulfide isomerase A6 (PDIA6) limits the IRE1α activity by binding to cysteine 148 of IRE1α, which maintains it within a physiologically appropriate range [33]. As a direct target of IRE1, a single mRNA that encodes X-box binding protein 1 (XBP1) undergoes non-conventional splicing. As a result, a translational frame-shift is generated and the spliced *Xbp1* (XBP1s) isoform is produced [25,36]. XBP1s activates the expression of a subset of UPR-related genes, involving molecular chaperones, foldases, and components of ERAD, all of which together relieve ER stress and restore proteostasis [55,56]. In addition, XBP1s enhances membrane expansion and lipid synthesis [23].

Under sustained ER stress, the RNase activity of IRE1 can efficiently degrade many ER-bound mRNAs, including IRE1 itself, through regulated IRE1-dependent decay (RIDD), which is a conserved mechanism via which IRE1 cleaves transcripts possessing the consensus sequence, CUGCAG with a stem-loop structure [57,58,59,60,61]. For example, IRE1 in a hyperactive state under prolonged ER stress cleaves microRNAs that normally repress pro-apoptotic targets, which in turn promotes programmed cell death [62,63,64]. Intriguingly, IRE1 also mediates signaling crosstalk with stress signaling pathways involving c-Jun N-terminal kinase (JNK) and nuclear factor κB (NF-κB) via the association with adaptor proteins, suggesting the role of IRE1 in the formation of a signaling platform termed as the UPRosome [65,66].

### 2.3. ATF6

ATF6 is an ER-resident transmembrane protein possessing a basic leucine zipper-binding domain (bZIP) transcription factor within its cytosolic domain. Interestingly, under normal condition, ATF6 exists as a monomer, dimer, or oligomer via intra- and intermolecular disulfide bonds formed between the two conserved cysteine residues in its luminal domain [67]. Upon ER stress, ATF6 is released from BiP and translocates to the Golgi apparatus, where it is processed by the Golgi enzymes, site 1 protease (S1P) and S2P, leading to the transport of its cleaved cytosolic p50 fragment into the nucleus for the regulation of transcription. Additionally, it was shown that, under ER stress conditions, protein disulfide isomerase A5 (PDIA5) rearranges the disulfide bond in ATF6, thereby leading to the activation of ATF6 [34]. The cytosolic p50 fragment induces the expression of genes such as *chop* and *Xbp1* to increase the capacity of the ER to resolve ER stress, as well as genes required for ERAD [68,69,70,71]. Intriguingly, XBP1s and ATF6 can heterodimerize and also induce the expression of genes involved in ERAD [70,72]. However, ATF6 was also demonstrated to drive lipid biosynthesis and ER expansion in the absence of XBP1s [73].

## 3. Protein Quality-Control Machineries of the ER: Autophagy

Macroautophagy (hereafter autophagy) is a process via which cytoplasmic constituents involving proteins, aggregates, and whole organelles are degraded by the lysosome. While basal autophagy degrades long-lived proteins and organelles, autophagy can also be facilitated by stresses, including nutrient starvation and energy depletion. Autophagy begins with engulfment and sealing off these cytoplasmic constituents within double-membraned vesicles, referred to as autophagosomes. The formation of autophagosomes is regulated by the Unc-51-like kinase (ULK) complex, which consists of ULK1, autophagy-related protein 13 (ATG13), ATG101, and focal adhesion kinase (FAK) family kinase-interacting protein of 200 kDa (FIP200) [74]. Nucleation of the phagophore membrane is accomplished by a class III phosphatidylinositol 3-kinase (PI3K) complex that comprises PI3K catalytic subunit type 3 (PIK3C3), autophagy/beclin1 regulator 1 (AMBRA1), Vps15, beclin1, and ATG14-like protein. Elongation of the phagophore is mediated by two ubiquitin-like conjugations [75,76,77]. The first conjugation system that consists of ATG4, ATG7, and ATG3 mediates the conjugation of phosphatidylethanolamine to free microtubule-associated protein 1A/1B light chain 3 (MAP1LC3, also referred to LC3-I) in the cytoplasm, resulting in the formation of lipidated LC3 (LC3-II). The second conjugation system, ATG12/ATG5/ATG16 complex targets LC3 to the expanding phagophore membrane. The phagophore membrane eventually forms the autophagosome, which in turn fuses with lysosome, resulting in the formation of autolysosome. The ULK complex is controlled negatively or positively by mechanistic target of rapamycin complex 1 (mTORC1) or by 5′ AMP-activated protein kinase (AMPK), respectively. The activities of ULK1, mTORC1, and AMPK are tightly controlled on lysosomal membranes, which is essential for maintaining energy and amino-acid homeostasis. Under normal conditions, mTORC1 phosphorylates ULK1, which attenuates autophagosomal maturation and lysosomal activity, thereby leading to the downregulation of autophagy [78,79]. Additionally, mTORC1 downregulates autophagy via phosphorylation of AMBRA1 at serine 52, [80]. The mTORC1-mediated phosphorylation of AMBRA1 attenuates ULK1 ubiquitination by tumor necrosis factor (TNF) receptor-associated factor 6 (TRAF6), which leads to the destabilization and inactivation of ULK1. However, under energy-deficient or amino-acid starvation conditions, ULK1 and AMPK rapidly switch off the activity of mTORC1 and subsequently induce autophagy. AMPK phosphorylates ULK1, which is pivotal for the activity of ULK1 [81,82,83,84]. Phosphorylated ULK1 subsequently phosphorylates Raptor, resulting in the inhibition of association between Raptor and mTORC1 substrates and subsequent downregulation of mTORC1 signaling [85]. Furthermore, AMPK also phosphorylates Raptor and in turn inhibits mTORC1 [86]. Interestingly, autophagy began being suggested as one of the protein quality-control machineries of the ER to clear misfolded proteins and/or protein aggregates in the ER [14,18,87,88].

Transmembrane ER-resident UPR sensors are linked to autophagy [87,89]. ER stress downstream of hypoxia was demonstrated to induce ATF4- and CHOP-mediated upregulation of LC3 and ATG5 [90]. Furthermore, kinases, general control nonderepressible 2 (GCN2) and PERK, and transcription factors, ATF4 and CHOP, activate the transcription of a set of genes implicated in autophagy, involving LC3, ATG5, ATG3, ATG7, ATG10, beclin1, gamma-aminobutyric acid receptor-associated protein (GABARAP), p62, and neighbor of *BRCA1* gene 1 (NBR1) [91]. 

IRE1 associates with TRAF2 and induces the phosphorylation of apoptosis signal-regulating kinase 1 (ASK1), resulting in JNK phosphorylation, which phosphorylates B cell lymphoma 2 (Bcl-2). The phosphorylation of Bcl-2 results in its dissociation from beclin 1, which activates the beclin 1 and phosphatidylinositol 3-kinase (PI3K) complex, and subsequently, promotes autophagy [92,93]. Additionally, ER stress can modulate the activity of mammalian target of rapamycin complex 1 (mTORC1) as well as AMPK. Mechanistically, the PERK/ATF4 axis upregulates the expression of sestrin 2 and DNA damage-inducible transcript 4 (DDIT4), which inhibits the activity of mTORC1, resulting in autophagy [87]. Furthermore, ATF4-mediated upregulation of CHOP induces the upregulation of tribbles homolog 3 (TRB3), thereby leading to the decrease in AKT1 phosphorylation and the subsequent inhibition of mTORC1 [87].

Recently, ER-bound autophagy receptors that interact with cleaved LC3, LC3-I, were identified. The ER-bound autophagy receptors as ER-resident proteins are either transmembrane proteins or ER membrane-anchored proteins, exposing at least one LC3-interacting region (LIR) motif to the cytoplasm. The association of ER-bound autophagy receptors with LC3 leads to the ER sequestration into autophagosomes, subsequently resulting in ER-phagy, selective autophagy for the ER. There are three ER-bound autophagy receptors for LC3, involving family with sequence similarity 134 member B (FAM134B), reticulon 3 (RTN3), and Sec62 [94,95]. RTN3 and FAM134B possess a reticulon homology domain (RHD), via which RTN3 and FAM134B are anchored to the ER membranes. Intriguingly, RHD strikingly mediates ER fragmentation, thereby facilitating ER-phagy [94,95,96]. Additionally, Sec62 is specifically involved in ER-phagy during the recovery from ER stress, and clears ER fragments enriched in redundant ER chaperones [97].

The link between autophagy and protein quality control of the ER remains largely unknown, especially at a detailed mechanistic level. However, autophagy of the ER, particularly ER-phagy, began emerging as an effector pathway of the ER stress response signaling pathway and is suggested to play a pivotal role in the maintenance of ER homeostasis and subsequent proteostasis.

## 4. Protein Quality-Control Machineries of the ER: ERAD (ER-Associated Degradation)

**Figure 2:**
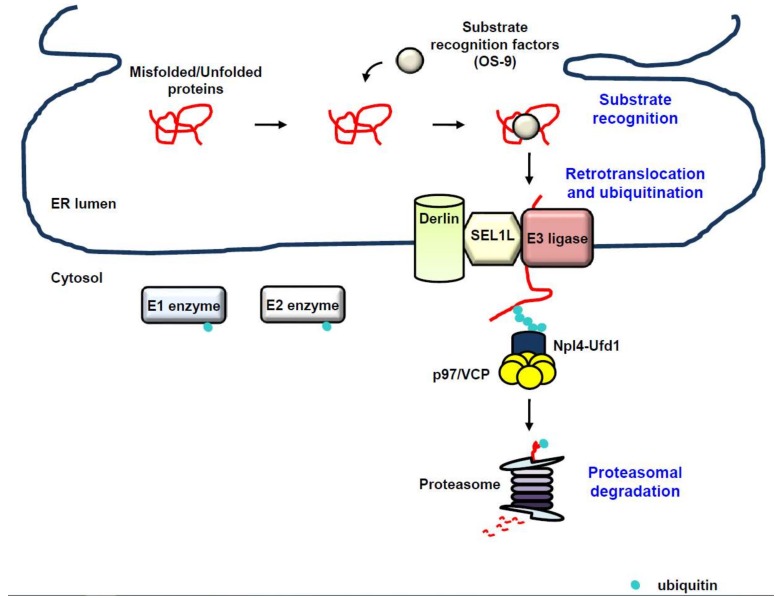
ER-associated degradation (ERAD). ERAD is the conserved protein quality-control machinery of the ER for eliminating misfolded, unassembled, or tightly regulated proteins via the cytosolic ubiquitin proteasome system (UPS). Substrate recognition. Proteins failing to acquire their adequate conformation are recognized by substrate recognition factors such as osteosarcoma 9 (OS-9) for ERAD (substrate recognition). Retrotranslocation and ubiquitination. Once the substrate is recognized, it is subject to retrotranslocation and ubiquitination. Recognition of the substrate accelerates the assembly of the retrontranslocon and the initiation of substrate polyubiquitination via the sequential enzymatic system of ubiquitin E1-activating, ubiquitin E2-conjugating, and ubiquitin E3 ligase. Proteasomal degradation. The retrotranslocated substrate is eventually guided to the proteasome, thereby leading to its degradation (proteasomal degradation). Black arrow, facilitation; red line, misfolded/unfolded protein.

Despite the highly coordinated machinery of the ER for co- and post-translational folding and maturation of the polypeptide chain, the process of protein folding and maturation is not perfect and is error-prone, which can compromise cellular and organelle homeostasis, and therefore, must be eliminated [21,22]. ERAD is the conserved protein quality-control machinery of the ER for eliminating misfolded or unassembled proteins via the cytosolic ubiquitin proteasome system (UPS) [14,15,16,17]. Additionally, growing evidence suggests that ERAD also targets correctly folded proteins, involving metabolically controlled enzymes, plasma membrane transporters, and transcription factors, thereby fine-tuning cellular homeostasis [98,99]. ERAD is an elaborate and multi-step process that recognizes, extracts from the ER, and ubiquitinates proteins for degradation by the cytosolic 26S proteasome [10,17,100,101,102] (Figure 2). Whereas integral membrane proteins can be easily directed to the UPS that is located in the cytoplasm or on the cytoplasmic face of the ER membrane, luminal proteins must be extracted from the ER for ERAD. Intriguingly, a failure of the ERAD to clear misfolded or unassembled proteins results in the accumulation of these abnormal proteins, which is closely associated with a variety of human diseases, involving cancer, neurodegeneration, and metabolic diseases [103]. 

### 4.1. Recognition

Substrate recognition is the commitment step for the ERAD process and can be conducted by molecular chaperones and chaperone-like lectins [13]. A large number of proteins synthesized in the ER are co-translationally modified by the attachment of high-mannose “core” glycans, possessing the structure Glc_3_Man_9_GlcNAc_2_ (Glc: glucose, Man: mannose, GlcNAc: *N*-acetylglucosamine), to consensus asparagine residues within canonical *N*-glycosylation sites (NxS/T), which plays a pivotal role in monitoring conformational maturation, directing correctly folded proteins to ER exit, and directing misfolded proteins to ERAD [104]. The lectin-type chaperone, calnexin or calreticulin, binds to Glc_1_Man_9_GlcNAc_2_ produced by deglucosylation of the core glycans, thereby facilitating the folding of immature glycoproteins [104]. Incompletely folded proteins are subject to reglucosylation by uridine diphosphate (UDP)-glucose:glycoprotein glucosyltransferase (UGGT) and undergo further rounds of folding via reassociation with calnexin or calreticulin. In contrast, further demannosylation from *N*-glycan blocks additional binding of the glycoproteins to calnexin or calreticulin, allowing ER exit of the proteins [105,106]. Therefore, terminally misfolded proteins must escape from the calnexin/calreticulin cycle, which is regulated by mannosidases that progressively eliminate terminal mannose residues from core glycans, thereby leading to association with mannose-specific lectins for ERAD [105,107]. ER mannosidase I (ERManI) [108,109], ER degradation-enhancing α-mannosidase-like protein 1 (EDEM1) [110,111], EDEM3 [112,113], or Golgi-resident mannosidase α class 1C member 1 (Man1C1) [114] trims terminal mannoses, which enables ERAD to discriminate misfolded proteins from their maturation-competent counterparts. Osteosarcoma 9 (OS-9) and xanthosine 5′-triphosphate (XTP)-B/Erlectin, known as ER-resident lectins, eventually recognize these mannose-trimmed misfolded proteins, subsequently recruiting them to the protein penetration channel, retrotranslocon, for ERAD [115,116,117]. Interestingly, non-glycosylated proteins can be targeted to ERAD. The non-lectin chaperone BiP interacts with non-glycosylated proteins for targeting to ERAD [118,119]. Furthermore, EDEM1 or PDI is involved in the targeting of non-glycosylated proteins to ERAD [120,121].

### 4.2. Retrotranslocation and Ubiquitination

Since the ER lumen does not contain any components involved in UPS, such as E1 ubiquitin-activating enzyme, E2 ubiquitin-conjugating enzyme, or the proteasome, the energy-dependent protein extraction step across the ER membrane back into the cytoplasm, known as dislocation or retrotranslocation, is required [122]. Intriguingly, the processes of retrotranslocation, ubiquitination, and proteasomal degradation of ERAD substrates should be tightly coupled, since many ERAD substrates are highly hydrophobic and easily aggregate in an aqueous environment. Remarkably, the ER membrane-embedded E3 ubiquitin ligases were demonstrated to comprise part of the retrotranslocon [123].

The p97/valosin-containing protein (VCP), a homohexameric enzyme, is a member of the type II AAA+ protein family of ATPases. The p97/VCP enzyme consists of two AAA domains, D1 and D2, that are assembled in a head-to-tail manner, an N-terminal domain that plays a role in substrate recognition, and a C-terminal domain that interacts with a large number of adaptors, which explains the diversity of p97/VCP interacting partners [124,125,126,127]. The p97/VCP enzyme plays an essential role in the retrotranslocation of nearly all ERAD substrates via coupling ATP hydrolysis to unfolding of ERAD substrates, with the assistance of cofactors recruited through p97/VCP-binding domains, involving p97/VCP-interacting motif (VIM), p97/VCP-binding region (VBR), and the SHP (BS1, binding segment 1) box [125]. Most p97/VCP cofactors also possess ubiquitin-binding domains (UBDs) and interact directly with ubiquitinated substrates. The p97/VCP enzyme, with the assistance of its cofactors, nuclear protein localization protein 4 (Npl4) and ubiquitin fusion degradation protein 1 (Ufd1), cooperatively produces a driving force for the retrotranslocation of substrates for ERAD [128,129]. The substrate for ERAD is slightly exposed to the ER surface through the retrotranslocon, subject to polyubiquitination mediated by E3 ubiquitin ligase, and further retrotranslocated by the p97/Npl4/Ufd1 complex, which is able to recognize the polyubiquitinated substrate, indicating that polyubiquitination plays a key role in the p97/VCP-mediated substrate extraction. To summarize, the processes of retrotranslocation and E3 ubiquitin ligase-mediated polyubiquitination should be tightly coupled and p97/VCP provides a platform for the various factors involved in ERAD to regulate ubiquitination at sites of retrotranslocation [100,130,131,132,133,134].

A dozen ERAD E3 ubiquitin ligases were identified to date. Several ERAD E3 ubiquitin ligases are transmembrane proteins, involving hydroxymethylglutaryl reductase degradation protein 1 (HRD1), glycoprotein 78 (gp78), membrane-associated really interesting new gene (RING) finger protein 6 (MARCH6), and RING finger protein 5 (RNF5) [100,135,136,137,138,139,140,141]. Additionally, cytoplasmic E3 ubiquitin ligases, involving C terminus of Hsp73-interacting protein (CHIP), parkin, and Skp, Cullin, F-box containing complex (SCF) complexes with the F-box proteins Fbx2, Fbx6, and beta-transducin repeat containing proteins 1 and 2 (β-TrCP1/2), SMAD ubiquitination regulatory factor 1 (Smurf1), and neuregulin receptor degradation pathway protein 1 (Nrdp1)/fetal lever ring finger (FLRF), were demonstrated to be involved in ERAD [142,143,144,145,146,147,148]. E3 ubiquitin ligases for ERAD may accomplish polyubiquitination of substrates by cooperating with other E3 ubiquitin ligases, by attaching ubiquitin at multiple sites of a substrate via E4 ubiquitin ligase-mediated extension after initial monoubiquitination, or by sequential rounds of ubiquitination and deubiquitination, raising the possibility that various strategies evolved for the optimal efficiency of ERAD [149,150,151,152,153].

HRD1 plays a role in the degradation of not only glycosylated but also non-glycosylated ERAD substrates. HRD1 generally ubiquitinates glycosylated substrates in a suppressor/enhancer of Lin12-like (SEL1L)-dependent manner. SEL1L is not only required for the transfer of substrates to HRD1, but also essential for the stabilization of HRD1, suggesting that SEL1L is important for the recruitment, retrotranslocation, and ubiquitination of ERAD substrates. [116,154,155,156,157]. SEL1L recruits recognition factors for luminal substrates, including OS-9, XTP3-B, EDEMs, ERdj5, and PDI to the retrotranslocon [101,158]. Additionally, SEL1L plays a role as a scaffold in the formation of a complex with Derlin-1, Derlin-2, ancient ubiquitous protein 1 (AUP1), ubiquitin regulatory X (UBX) domain-containing protein 8 (UBXD8), and VCP-interacting membrane protein (VIMP) [116,117,159,160,161,162,163], which subsequently recruits the p97/VCP, resulting in the substrate retrotranslocation. Mammalian cells have three Derlins: Derlin-1, -2, and -3. As a part of the retrotranslocon channel [164,165], Derlins bind to substrates and target them to p97/VCP, as well as to E3 ubiquitin ligases [123]. Recently, it was shown that, as chaperones, Ubiquilin and Bcl-2-associated athanogene 6 (Bag6) play important roles as holdases for retrotranslocated proteins prior to the HRD1-mediated ERAD process [166,167].

The endogenous substrates for SEL1L/HRD1-mediated ERAD were identified. IRE1, p53, Fas, B-cell development-specific pre-B cell receptor (pre-BCR), peroxisome proliferator activated receptor γ coactivator-1 β (PGC1β), B-lymphocyte-induced maturation protein 1 (BLIMP1), NF-E2-related factor 2 (NRF2), and pro-opiomelanocortin (POMC) were identified as substrates for SEL1L/HRD1-mediated ERAD using cell-type specific SEL1L- or HRD1-deficient mouse and cellular models [157,168,169,170,171,172,173]. It was demonstrated that mice with HRD1- or SEL1L-deficient adipocytes show postprandial hyperlipidemia [169]. Additionally, mice with SEL1L deficiency in arginine vasopressin (AVP) neurons were demonstrated to develop polyuria and polydipsia [171], suggesting the pathophysiological importance of the SEL1L/HRD1 ERAD protein quality-control machinery in health and disease. 

Glycoprotein 78 (Gp78) is another major E3 ubiquitin ligase for ERAD in mammalian cells [137]. In a similar manner to HRD1, Gp78 recruits UBXD2 or UBXD8, thereby cementing p97/VCP at the ER membrane. Alternatively, Gp78 can directly associate with p97/VCP through its VIM domain [132]. Interestingly, Gp78 also functions as an E4 ubiquitin ligase in a cooperative manner with E3 ubiquitin ligases [149]. For instance, Gp78 serves as an E4 ubiquitin ligase for RNF5-mediated ubiquitination of cystic fibrosis transmembrane conductance regulator (CFTR)∆F508 in ERAD [139,149].

Cytosolic E3 ubiquitin ligase, CHIP, also acts as an E3 ubiquitin ligase for the ubiquitination of CFTR for ERAD [174]. Interestingly, CHIP functions sequentially with RNF5 to degrade misfolded CFTR [139]. While CHIP acts on CFTR∆F508 post-translationally, RNF5 recognizes folding defects in CFTR∆F508 coincident with translation.

Interestingly, it was demonstrated that ERAD E3 ubiquitin ligases may ubiquitinate other ERAD components involved in the recruitment of p97/VCP or possessing UBDs such as Gp78, AUP1, ubiquitin-associated (UBA)-domain-containing protein 2 (UBAC2), and UBXD8 [100,175,176]. Furthermore, ERAD E3 ubiquitin ligases ubiquitinate each other, which forms a negative feedback loop and fine-tunes the ERAD process [101,149,150,177,178].

### 4.3. Proteasome-Mediated Degradation

The p97/VCP enzyme plays an essential role in linking retrotranslocated substrates to cytoplasmic cofactors involved in further processing of substrates, suggesting that p97/VCP is closely associated with the proteasome-mediated degradation of substrates [125]. The deglycosylating enzyme, *N*-glycanase 1 (NGly1), localized in the cytoplasm is recruited to retrotranslocon complexes via direct binding to p97/VCP and cleaves *N*-linked glycans from retrotranslcated substrates for ERAD [179,180].

Deubiquitinating enzymes (DUBs), including ovarian tumor family of deubiquitinating enzyme 2 (OTUD2, also known as YOD1), valosin-containing protein p97/p47 complex-interacting protein p135 (VCIP135), ubiquitin-specific protease 13 (USP13), and Ataxin-3, associate with p97/VCP either directly or indirectly and deubiquitinate ERAD substrates [181,182,183]. YOD1 was suggested to cooperate with p97/VCP, resulting in the efficient retrotranslocation of ubiquitinated substrates. Interestingly, it was demonstrated that impairment of p97/VCP-associated deubiquitination or expression of dominant-negative YOD1 attenuates retrotranslocation and degradation of ERAD substrates, whereas expression of DUBs restores them [181], indicating that sequential rounds of ubiquitination and deubiquitination are essential for efficient ERAD process. The role of ataxin-3 in ERAD remains elusive. It was suggested that ataxin-3 acts after retrotranslocation of ERAD substrates, supporting the degradation of ERAD substrates [183]. Alternatively, ataxin-3 was shown to remove the ubiquitin from ERAD substrates, thereby increasing the half-life of protein and providing time for folding of ERAD substrates [184]. It was shown that USP13 associates with p97/VCP, Ufd1, Npl4, and UBXD8, and that depletion of USP13 leads to the accumulation of ERAD substrates [182]. 

Retrotranslocated substrates for ERAD should be rapidly degraded to prevent the substrates from aggregating in the cytoplasm. A chaperone complex composed of Bag6/ ubiquitin-like protein 4A (Ubl4A)/transmembrane domain recognition complex 35 (Trc35)- cochaperone small glutamine-rich TPR-containing protein α (SGTA) is known to be involved in this process [185]. Bag6 forms a homooligomer through a proline-rich domain. Interestingly, the proline-rich domain is sufficient for binding of Bag6 to the hydrophobic segments of misfolded proteins, which maintains misfolded proteins in a soluble state [186]. Furthermore, the holdase activity of Bag6 is also essential for maintaining retrotranslocated substrates in a competent state for proteasomal degradation [167]. Bag6 also interacts with the proteasome, as well as the adaptor proteins of proteasome, which transfers retrotranslocated substrates to proteasome for degradation.

## 5. Protein Quality-Control Machineries of the ER and Cancer

The ability of tumor cells to response to various stresses and subsequently to overcome the stresses depends on the capacity of tumor cells to successfully activate appropriate adaptive pathways [65,187]. In the course of malignant transformation, tumor cells are exposed to not only extrinsic stresses such as nutrient deprivation, accumulation of acidic waste, and hypoxia, but also intrinsic stresses such as alteration in chromosome number, activation of oncogenes, inactivation of tumor-suppressive genes, and accelerated secretion, subsequently triggering exacerbated protein synthesis, which results in a cellular state of ER stress and in turn activates protein quality-control machineries of the ER [188,189,190,191]. Intriguingly, chronic UPR at later stages was demonstrated to lead to the adaptation of the tumor to extrinsic and intrinsic perturbations and confer resistance to ER stress-induced apoptosis on tumor, while transient UPR at early stages of tumorigenesis often impedes tumor progression [192]. Protein quality-control machineries of the ER were demonstrated to be involved in diverse human cancers. In fact, protein quality-control machineries of the ER could play a pivotal role in the control of tumor progression, angiogenesis, and metastasis, affect tumor microenvironment involving immune cells and endothelial cells, and modulate the efficacy of therapies for cancer [4].

### 5.1. Protein Quality-Control Machineries of the ER and Tumor Progression

PERK was demonstrated to associate with the progression of various tumors. Depletion of PERK was shown to facilitate tumor progression [193,194]. The ATF4/CHOP axis promotes protein synthesis and subsequently accelerates ROS production, while the treatment of antioxidants and depletion of RPL24 reduces apoptosis by decreasing ROS production and protein synthesis, indicating that PERK is involved in tumor suppression [49]. On the contrary, PERK was demonstrated to accelerate tumor growth by stabilizing NRF2, modulating redox homeostasis, and regulating lipid biosynthesis [193,195,196,197,198,199]. Furthermore, PERK is closely linked to angiogenesis. For supply of sufficient oxygen and nutrients, growing cancer cells produce pro-angiogenic factors to initiate vascularization. PERK promotes the expression of the vessel growth and stabilization factors, vascular endothelial growth factor and type I collagen inducible protein (VCIP) and platelet derived growth factor receptor B (PDGFRB) [194]. Additionally, PERK-mediated upregulation of vascular endothelial growth factor (VEGF), fibroblast growth factor 2 (FGF2), and interleukin-6 (IL-6), and downregulation of anti-angiogenic cytokines remarkably facilitate tumor growth [199].

IRE1 is also associated with tumor progression. Mutated forms of IRE1 were shown to promote tumor progression, even though some of these mutants still had intact kinase and endoribonuclease activity [62,200,201]. Activated JNK by IRE1 not only suppresses anti-apoptotic Bcl-2 activity, but also accelerates the action of pro-apoptotic Bcl-2 interacting mediator of cell death (BIM), resulting in cell death [4]. Furthermore, RIDD was shown to activate pro-apoptotic caspase-2 in mouse embryonic fibroblasts (MEFs) [63,64]. In contrast, it was demonstrated that IRE1-mediated activation of signal transducer and activator of transcription 3 (STAT3) and NF-κB upregulates the expression of anti-apoptotic proteins, involving Bcl-2 family members, inhibitor of apoptosis protein (IAP), myeloid cell leukemia sequence 1 (Mcl-1), and caspase-8 inhibitor c-FLICE-like inhibitor protein (c-FLIP) [202]. Additionally, the IRE1/XBP1 axis is associated with poor prognosis in pre-B acute lymphoblastic leukemia and glioblastoma [203,204,205,206,207]. The IRE1/XBP1 axis also facilitates angiogenesis. XBP1s was demonstrated to associate with hypoxia-inducing factor 1α (HIF1α), a key regulator of VEGF in triple negative breast cancer (TNBC) cells, thereby promoting angiogenesis [208].

The role of ATF6 in tumor progression began being elucidated. Depletion of ATF6 was shown to induce the death of multiple myeloma cell lines [209]. On the contrary, the overexpression of active ATF6 decreases an anti-apoptotic protein, Mcl-1, thereby mediating apoptosis [210]. Although the role of ATF6 in tumorigenesis remains elusive, it was recently demonstrated that upregulated ATF6 in leukemia cells confers resistance to imatinib and depletion of ATF6 restores sensitivity to imatinib [34].

ERAD in tumor progression remains largely unknown. However, it began emerging that the high degrees of cell growth, as well as high rates of mutation in cancer cells, lead to an accumulation of unfolded and/or misfolded proteins, thereby leading to the activation of the ERAD process [211,212]. In colorectal cancer, SEL1L is upregulated in adenoma and adenocarcinoma cells [213]. On the contrary, higher expression of SEL1L in pancreatic cancer cells leads to not only gap 1 (G1) phase cell-cycle arrest via the induction of a phosphatase and tensin homolog (PTEN) but also the reduction in invasiveness by modulating genes related to cell–matrix interactions [214,215].

Gp78 was shown to be linked with several types of cancers [216,217,218]. Colorectal cancer patients with upregulation of Gp78 have poor survival and high recurrence of cancer, indicating that Gp78 is closely linked to increased risk of cancer with lower survival rate [219,220,221].

To summarize, the involvement of protein quality-control machineries of the ER in tumor progression is a matter of debate. Therefore, to clarify the role of protein quality-control machineries of the ER in cancer pathogenesis, it is pivotal to elucidate the mechanism via which protein quality-control machineries of the ER are involved in tumor progression within a specific tumor context, as well as the changes in the expression of components involved in protein quality-control machineries of the ER in tumor cells.

### 5.2. Protein Quality-Control Machineries of the ER and Metastasis

Metastasis is a complicated process in which cancer cells migrate from the original tumor site, infiltrate the extracellular matrix (ECM) and stromal cell layers, penetrate the lymphatic circulatory systems, colonize foreign tissues, and grow into a new tumor mass [187,191,222,223]. The PERK/ATF4 axis was demonstrated to activate lysosome-associated membrane protein 3 (LAMP3), resulting in the metastasis of breast cancer cells [224,225]. Additionally, the upregulation of ATF4 in esophageal squamous carcinoma leads to metastasis through the regulation of matrix metalloproteinases [226]. Furthermore, the PERK/ATF4 axis was shown to be potentially linked to the expression of genes involved in epithelial-to-mesenchymal transition (EMT) [187].

The IRE1/XBP1 axis is also involved in metastasis. It was demonstrated that XBP1s forms a transcriptional complex with HIF1α, which upregulates the expression of HIF1α targets such as pyruvate dehydrogenase kinase 1 (PDK1) and glucose transporter 1 (GLUT1), thereby driving TNBC tumorigenicity and invasiveness [208]. Surprisingly, while IRE1 in malignant glioma is positively associated with the upregulation of proangiogenic factors such as VEGF-A, IL-1β, IL-6, and IL-8, it induces significant downregulation of proteins linked to mesenchymal differentiation and glioma invasiveness, such as secreted protein acidic and rich in cysteine (SPARC), decorin, and thrombospondin-1 [227,228], strongly suggesting that a comprehensive analysis of the IRE1/XBP1 axis is required to determine the relationship between invasiveness and angiogenesis.

The role of Gp78 in metastasis is largely unknown. It was shown that Gp78 inversely correlates with E-cadherin in patients with bladder carcinomas, as well as gastric cancers [229,230,231]. Furthermore, Gp78-mediated ERAD of metastasis suppressor protein Kangai1 (KAI1) was demonstrated to promote metastasis, raising the possibility of the involvement of Gp78 in metastasis [232,233].

### 5.3. Protein Quality-Control Machineries of the ER and Cancer Immunogenicity

The tumor microenvironment is a complicated environment consisting of stromal cells such as fibroblasts and endothelial cells and infiltrating immune cells such as cluster of differentiation 8 (CD8) T cells, regulatory T cells (Tregs), myeloid-derived suppressor cells (MDSCs), and dendritic cells (DCs). Intriguingly, the relationship between ER stress response in tumor-associated immune cells and tumor progression began being elucidated [187]. The ER stress response driven by hyperactivated XBP1 facilitates neutrophil-infiltrating acute lung injury [234]. Furthermore, sustained activation of the IRE1/XBP1 axis was demonstrated in ovarian tumor-infiltrating DCs [235]. Intriguingly, the ovarian tumor-infiltrating DCs were shown to facilitate ROS production and consequential disruption of ER homeostasis, resulting in the control of anti-tumor immunity. Additionally, the status of ROS-promoted lipid peroxidation was suggested as a biomarker of disease recurrence in breast cancer patients [236]. Interestingly, tumor-infiltrating DCs deficient in XBP1 acquire immunostimulatory and anti-tumoral characteristics in vivo [237,238,239]. Pharmacological inhibition of IRE1 in bone-marrow-derived macrophages stimulated by IL-6 and IL-4 was shown to inhibit macrophage-mediated cell invasion in vitro [240]. Additionally, pharmacological induction of ER stress upregulates the lectin-type oxidized low-density lipoprotein (LDL) receptor-1 (LOX-1) in neutrophils and induces transformation of neutrophils into immunosuppressive cells [241,242], suggesting that the IRE1/XBP1 axis controls tumor-associated myeloid cells.

CHOP was shown to be upregulated in tumor-infiltrating MDSCs [243]. Tumor-infiltrating MDSCs lacking CHOP show decreased immunosuppressive activity toward T cells. Furthermore, the ATF4/CHOP axis in tumor-infiltrating MDSCs accelerates apoptosis through death receptor 5 (DR5) and caspase-8 activation [244], suggesting that UPR activation plays a key role in modulating tumor-associated immune responses.

Through a process of “transmissible ER stress”, ER stress enables tumor cells to secrete several factors that promote macrophage activation and induce a pro-inflammatory response in the microenvironment of tumors [245]. This process not only attenuates the antigen-presenting capacity of bone-marrow-derived DCs and attenuates T-cell proliferation, but also facilitates the upregulation of immunosuppressive molecules [246], suggesting the involvement of ER stress signaling in immune escape. However, ER stress also facilitates immunogenic cell death (ICD) and anti-tumor immunity [247]. ICD promotes the release of damage-associated molecular patterns (DAMPs), involving surface exposure of calreticulin, ATP secretion, and passive release of high-mobility group box 1 (HMGB1), raising the possibility that, as signals of danger, DAMPs facilitate anti-tumor immunity [192,248,249]. PERK was demonstrated to be associated with the exposure of calreticulin in non-small-cell lung carcinoma (NSCLC) and to be positively associated with ICD and anti-tumor immunity [250]. Intriguingly, it was shown that photodynamic therapy activates PERK signaling and increases the surface exposure of calreticulin, as well as ATP secretion, in human bladder carcinoma, resulting in the engulfment of cancer cells by DCs [251]. Furthermore, radiation and anthracycline treatment lead to lethal ER stress, the excessive activation of UPR, and an increase in the level of cytosolic Ca^2+^, resulting in the activation of inflammasome and subsequent ICD [63,252].

### 5.4. Protein Quality-Control Machineries of the ER and Therapies for Cancer

The role of protein quality-control machineries of the ER in the course of tumorigenesis and their clinical significance, as well as prognostic implications, still remain a matter of debate [253]. However, accumulating evidence indicates that protein quality control in the ER represents a key process in the control of tumor development, stage progression, and resistance to therapies for cancer. Intriguingly, it was suggested that protein quality-control machineries of the ER may serve as tools for patient stratifications, since the activity of specific branches of the protein quality-control machineries of the ER began emerging as a predictive biomarker for prognosis. Furthermore, strategies to inhibit or activate protein quality-control machineries of the ER began emerging as new pharmacological tools for cancer treatment. In fact, a large number of anticancer drugs promote UPR activation, resulting in the development of chemosensitivity or chemoresistance in a context-dependent manner [140,187,254,255,256,257]. Furthermore, targeting of UPR of the ER itself in cancer cells was demonstrated to inhibit survival or promote cell death [192,258,259]. Targeting the PERK/eIF2α axis accelerates cell death of therapy-resistant colon carcinoma cells and hypoxic glioblastoma [260], raising the possibility that a combination of cancer therapy and UPR targeting may be a promising strategy for cancer treatment. 

Autophagy, one of the protein quality-control machineries of the ER, is closely associated with therapeutic resistance. It was demonstrated that an increase in PERK-mediated autophagy develops vemurafenib resistance in melanoma [254]. Intriguingly, simultaneous inhibition of B-rapidly accelerated fibrosarcoma (BRAF) (V600E) and PERK in melanoma increases chemosensitivity, suggesting that the balance between autophagy mediated by UPR and chemotherapy is essential for the overcoming of chemoresistance. Additionally, autophagy induced by the IRE1/JNK pathway is closely linked to sorafenib resistance in hepatocellular carcinoma cell lines [261,262].

As already mentioned, since protein quality control in the ER is closely associated with the functions of tumor-associated immune cells, and subsequently, pro-tumor or anti-tumor immune responses, it is essential to consider UPR-targeting therapies in immune cells. Silencing of *Ire1* or depletion of Xbp1 transforms DCs into immunostimulatory cells, resulting in survival through T-cell-mediated anti-tumor immunity [235]. Furthermore, transplanted MDSCs lacking DNA damage-inducible transcript 3 (*Ddit3*) exhibit enhanced antigen-presenting capacity and T-cell stimulatory effects [243]. Additionally, ERO1α was demonstrated to upregulate the expression of programmed death ligand 1 (PDL1) in TNBCs [263]. To summarize, the combination of strategies to modulate protein quality-control machineries of the ER with immunotherapies might be effective for cancer treatment. 

## 6. Conclusions and Future Perspectives

During malignant transformation, tumor cells are exposed to not only extrinsic stresses, but also intrinsic stresses, which trigger exacerbated protein synthesis, thereby leading to a cellular state of ER stress, and subsequently, activating protein quality-control machineries of the ER. The ability of tumor cells to overcome extrinsic and intrinsic stresses, and subsequently, survive largely depends on the capacity of tumor cells to successfully activate appropriate adaptive pathways to these stresses [65,187]. Intriguingly, it became evident that protein quality-control machineries of the ER not only act as a guardian of tumor progression in early stages, but also serve as a key player for maintenance of tumors under chronic ER stress. Furthermore, even though the pharmacological modulation of protein quality-control machineries of the ER is still a challenge, the modulation of protein quality-control machineries of the ER began being suggested as a potential way of treating malignancies and of overcoming intrinsic or acquired resistance of tumors to therapies. Therefore, to elucidate the precise molecular mechanisms and pathophysiological relevance of protein quality-control machineries of the ER remarkably contributes to novel therapeutic interventions for the treatment of cancer.
